# Resectional surgery for malignant disease of abdominal digestive organs is not surgery of the organ itself, but also that of the mesenteric organ

**DOI:** 10.1007/s10151-020-02197-7

**Published:** 2020-04-02

**Authors:** J. Bunni, J. C. Coffey, M. F. Kalady

**Affiliations:** 1 Department of Colorectal Surgery, Royal United Hospitals Bath NHS Foundation Trust, Combe Park, Bath, BA1 3NG UK; 2 University of Limerick Hospital Group, Limerick, Ireland; 3 Department of Colorectal Surgery, Digestive Disease and Surgery Institute, Cleveland Clinic, Cleveland, OH USA

**Keywords:** Mesentery, Complete mesocolic excision (CME), Central vascular ligation (CVL), D3 lymphadenectomy, Embryology, Ontogenetics, Colorectal cancer

## Abstract

Despite large strides in molecular oncology, surgery remains the bedrock in the management of visceral cancer. The primacy of surgery cannot be understated and a mesenteric (i.e. ontogenetic) approach is particularly beneficial to patients. Heald greatly advanced the management of rectal cancer with his description of the anatomical foundation of total mesorectal excision (TME), dramatically improving outcomes worldwide with this mesenteric-based approach. Moreover, complete mesocolic excision (CME) based on similar principles is becoming popular. Introduced by Hohenberger, CME resembles TME insofar as it emphasises strictly anatomical dissection along embryological planes to detach an intact (i.e. “complete”) mesentery with peritoneal envelope. CME also incorporates “central” vascular ligation (CVL) which broadly correlates with the “D3 lymphadenectomy” of Eastern literature. As many surgeons already practise anatomical and mesenteric-based surgery, it is unclear how the putative benefits of CME (including CVL) arise. Herein, we argue that these may relate to a more extensive resection of the mesentery, and thus mesenteric tumour deposits within the connective tissue lattice of the mesentery, and not necessarily the lymphadenectomy alone. We believe the connective tissue interface between the bowel wall and mesentery provides an alternative mode of spread of pathogenic elements. Whilst this remains a suggestion only, it would explain the histological independence of tumour deposits and why a greater mesenterectomy could be associated with benefits in survival. If this argument holds, it follows that resectional surgery for digestive organ malignancy is not surgery of the organ itself (or lymphatics only), but also that of the contiguous mesentery.

## Introduction

Colorectal cancer is the second commonest cause of cancer death in the UK, posing an enormous burden to patients, families and the economy in general. Despite many major advances in screening, treatment and heightened community awareness, 20% of patients still present with advanced disease. Whilst combination chemotherapy and multi-modal surgery (liver surgery, thoracic surgery, cytoreductive surgery and hyperthermic intraperitoneal chemotherapy) can extend life, stage IV disease does, in general, have a poorer long-term prognosis. In contrast, non-metastatic cases have an excellent probability of local control and cure, if careful case selection and high-quality surgery are employed.

Despite the era of biological chemotherapy, surgery remains the cornerstone of treatment in the management of visceral cancer. Recent reports indicate that 80% of all cases of cancer will need surgery and some several times [1]. There is no doubt as to the primacy of mesenteric-based resectional surgery in the management of colon and rectal cancer [2]. Increasing data now also support the adoption of mesenteric-based approaches in oesophageal, gastric and pancreatic cancer [3].

(“Bill”) Heald emphasised anatomical anatomical-based resection of the mesorectum as part of rectal resection in the surgical management of rectal cancer [4]. Prior to this, blunt extirpation of the rectum was the widespread (though not global) practice. Indeed in some domains, non-anatomical and non-mesenteric-based surgery is still practised, in particular in the emergency setting. Heald emphasised accessing the interface between the mesentery and fascia and separating both sharply. The zone was termed the “Holy Plane” and separation of components of the plane was later termed “mesofascial separation” [5]. Despite initial scepticism and sometimes incredulity at the dramatic improvements associated with total mesorectal excision (TME), it is now widely accepted as the gold standard approach.

The embryological and anatomical principles of TME were later extended to surgery for colon cancer, which had experienced a worse overall outcome compared with rectal cancer. Professor Werner Hohenberger described complete (i.e. intact) mesocolic excision and central vascular ligation (i.e. ligation of draining veins at their junction with the superior mesenteric vein) in 2009 [6]. His group demonstrated that adoption of CME was associated with a reduction in the local recurrence (LR) rate from 6.5 to 3.6% and increased-year cancer-related survival from 82.1 to 89.1% in Erlangen from 1978–1984 to 1995–2002.

What is the exact reason for these results?

## Discussion

Complete mesocolic excision is described by Hohenberger as “a surgical technique with sharp dissection of the visceral plane from the retroperitoneal one, aiming to avoid any breaching of the visceral fascia layer, which potentially may lead to tumour spread within the peritoneal cavity”.

Some surgeons would argue that CME is simply synonymous with “good surgery”. Data from West et al. [7] suggested, however, that only 50% of UK practice meets the aims of CME. This indicates that whilst CME may equate with “good surgery”, it is not standard practice. Increasingly, colorectal surgeons recognise the importance of mesofascial separation [5] and access the plane between mesentery and underlying fascia, when detaching the mesentery from the posterior abdominal wall. This is a requirement in good-quality minimally invasive colorectal surgery, especially with a medial-to-lateral approach. We believe that as a result of this, the number of surgeons practicing “CME” planar dissection is in fact higher than the data of West et al. (who analysed specimens from 1997 to 2002) might suggest.

Central vascular ligation (CVL) is, however, controversial, and arguably most UK, Irish and North American surgeons do not practise this when conducting a resection for tumours in the right colon. Most surgeons ligate the ileocolic artery close to its origin from the superior mesenteric artery and a handful more dissect out the superior mesenteric vein so as to ligate the ileocolic flush with the vein. The latter equates with a D2-type resection referenced in the Eastern literature. In CVL, the superior mesenteric vein is exposed near to where it passes posterior to the neck of the pancreas, and veins draining the right colon are ligated at this level. This approximates to the D3 lymphadenectomy described in Japanese literature [8].

Thus, since it appears that most surgeons do practice a mesenteric plane-based resection (albeit not all practice flush ligation with the superior mesenteric vein), especially in the laparoscopic era, could it be that the putative benefits of CME rest in the central vascular (D3) component? If so, what is the exact mechanism?

## Is there an anatomical basis to the potential benefits of central ligation of veins in the surgical management of colon cancer?

CME with CVL appear to be associated with a greater lymph node harvest and thus lymphadenectomy, when compared with conventional surgery. Caution must be applied to the term “conventional surgery”, as this cannot be precisely defined. This limitation is the key flaw in any appraisal of the differences between CME and non-CME approaches in the management of colon cancer.

Notwithstanding, it makes sense that CME and CVL would yield a larger volume mesentery than non-CME-based approaches. Hohenberger himself argues that by necessity, CVL is inextricably linked with CME, such that a “CME”, by definition, includes CVL to achieve a complete excision of the mesentery. We do however find that distinguishing the terms helps descriptively (especially with the use of immunofluorescence to target resection of specific lymph nodes only).

This would in turn support the contention that CME is associated with a higher lymph node yield. Lymph node harvest (i.e. lymphadenectomy) has multiple putative benefits [9]: (1) it is a specimen-based quality indicator of the surgical technique applied, (2) it facilitates prognostication, allowing administration of adjuvant therapy and (3) it may be therapeutic, by potentially interrupting cancer spread. Given the above, it is reasonable to link CME with improved outcomes.

But problems also arise with this argument. If one is performing embryological plane CME, then the quality of the surgery is already high and thus CVL adds little to this as a quality marker.

In terms of upstaging or the “Will Rogers phenomenon” it is unusual to have positive central (i.e. D3) nodes with negative peripheral (i.e. D1 or D2) nodes. “Skip” metastases probably account for 2% of lymphatic metastases at most. Therefore, the likelihood of correctly upstaging potentially missed nodes, and hence administering adjuvant chemotherapy, is low. The incidence of positive central nodes and peripheral nodes is also low.

A further problem lies in the fact that the mesentery does not stop at the central zone of the superior mesenteric vein. It continues proximally to the porta hepatis, thus explaining the occurrence of portal node metastases in patients with colorectal cancer [10]. This means that even though one may be achieving a potential benefit in excavating mesenteric tissue at the superior mesenteric vein, truly central vascular ligation would involve higher echelon nodes. Furthermore, the mesentery is fold shaped at its central zone. On the right side of the fold, veins draining the small intestinal mesentery, right and transverse mesocolon join the superior mesenteric vein. On the left side, the inferior mesenteric vein joins the superior mesenteric vein. The splenic vein is also anatomically close. The CVL component of CME addresses the right side of the central zone. A question arises as to whether one should also resect mesenteric tissue on the left (i.e. mesocolic side) of the central zone.

The concept of lymphadenectomy interrupting the dissemination of cancer is probably too simplistic and the process of metastasis is less likely to be Newtonian and ordered and more likely to be complex, random and multi-level in nature. Certainly, there is molecular evidence that metastasis and primary tumour growth are two autonomous processes [9]. It is also clear that circulating tumour cells can be identified in the circulation of patients at all stages of primary bowel cancer [11], and thus the original “seed and soil” hypothesis of Stephen Paget, the Halstedian basis of lymphadenectomy, is a gross oversimplification.

Despite this, a study by Chen and Bilchik [12] showed that in AJCC Stage III colon cancer, the 5-year overall survival increased from 67 to 90% (in patients with N1 disease) when either 1–10 lymph nodes or more than 40 nodes were removed, respectively. In addition, they showed that 5-year overall survival increased from 51 to 71% (in patients with N2 disease) when either 1–35 lymph nodes or when more than 35 nodes were removed, respectively. These improvements in overall survival are similar to results obtained when surgery is combined with optimal adjuvant treatment. Although the findings of Chen and Bilchik are correlated, and not definitive proof, they indicate that extensive lymphadenectomy may improve oncological outcome. We suspect that there still may be confounders in this study (for instance, some surgeons with higher lymph node yield may have performed better CME surgery) and that a more complete mesenterectomy is probably the reason.

It is important to note that the exact mechanisms by which metastases arise have not been fully elucidated. Increasing data indicate that modalities of spread other than haematogenous or lymphatic may be important [13, 14]. Mortality due to colorectal cancer is mainly due to the development of hepatic or systemic metastases. It is argued that hepatic metastases arise due to portal venous spread from a primary tumour. Systemic (non-portal) metastases are explained by both haematogenous and lymphatic spread, accessing the systemic circulation via the thoracic duct. Trans-coelomic spread (i.e. peritoneal metastases) are thought to arise more directly from locally advanced primary tumours (i.e. T4 tumours) shedding tumour cells which then spread inside the abdominal cavity via the redistribution phenomenon [15].

Isolated metastases (also termed tumour deposits) can be found within the mesentery [16] and are apparently (at least histologically) independent of blood vessels, lymphatic channels and nerves. Tumour deposits are potentially explained by recent findings related to the mesentery and intestine. Both are contiguous, which means they share histological elements at their interface [17]. Histological elements include connective tissue and it is now established that the connective tissue of the wall of the intestine is in continuity with that of the adjacent mesentery. The connective tissue platforms provide an alternative modality of spread of pathogenic elements [18]. Whilst this remains a suggestion only, it would explain the histological independence of tumour deposits. It would also explain why more extensive resection of the mesentery could be associated with putative benefits in survival. Furthermore, it is feasible that many mesenteric lesions that were interpreted as nodal metastases were in fact tumour deposits. If the last statement is correct, then what was originally interpreted as extensive lymphadenectomy was, in fact, more extensive resection of tumour deposits (i.e. not lymphatic metastases [19]).

Lord Moynihan famously remarked that “the surgery of malignant disease is not the surgery of organs, it is the anatomy of the lymphatic system”.

In general, whilst surgery is of course involved in the management of metastatic disease, the primary role and power of surgery relates to local control of the primary disease. It is increasingly recognised that extended mesenteric resection (a necessity with CME and CVL) confers a survival advantage that was earlier attributed to greater lymphadenectomy (in keeping with the suggestions of Moynihan, Jamieson and Dobson and many others).

## Conclusions

Although in the surgery of malignant disease a great emphasis is placed on lymphadenectomy, emerging data indicate that the benefits of mesenterectomy may extend beyond lymphadenectomy alone and relate to the resection of tumour deposits. If this holds, then the potential benefits of CVL (as part of CME) may not solely relate to extended lymphadenectomy, but also to extended resection of mesenteric tumour deposits. In keeping with this, we propose that Moynihan’s original assertion be edited to;

“Resectional surgery for malignant disease of abdominal digestive organs is not surgery of the organ itself, but also that of the mesenteric organ”.
